# Four Cases of Renal Calculi Treated Successfully by Lumbar Nephro-Lithotomy

**Published:** 1903-09

**Authors:** E. J. Trevor Jones


### FOUR CASES OF RENAL CALCULI TREATED SUCCESSFULLY BY LUMBAR NEPHRO-LITHOTOMY.

BY

E. J. TREVOR JONES, M.D. Brux., M.R.C.S., L.R.C.P. Lond.

---

THE early diagnosis and extraction of a stone from the kidney before suppurative changes have been induced by it is one of the great elements in the successful treatment of renal calculi. It is, however, a matter of common experience that exploratory incisions not infrequently fail in the detection of a calculus, which was supposed to be present from the character of the symptoms; but I have found one constant symptom in my cases—viz., hæmaturia—although we know this symptom may be entirely absent, and in each case it was more severe after violent exercise.

**Case 1.**—J. M., a collier, aged 24, came under my care in April, 1899. He had fixed renal pain on the left side, increased by abrupt bodily movement and exercise, radiating at times along the ureter to the groin and testicle, and sometimes a sharp stabbing pain was complained of on lumbar percussion. There was no history of definite renal colic. After a hard day's work underground he had noticed the urine of a dark colour, and on those occasions there was inability to sleep except on the painful side. These symptoms he had off and on for three years before he came to consult me. For the constant left lumbar pain he asked relief, and was admitted into the Aberdare Cottage Hospital. On May 25th, 1899, I cut down by the oblique lumbar incision, and on passing the finger to the pelvis I felt a stone. The kidney was incised along its convex border, my left finger and thumb controlling the hemorrhage. After a good deal of difficulty, I succeeded in extracting the stone. The hemorrhage was not severe. The kidney was now flushed out with 1 in 20 sterilised boracic solution, and a drainage tube passed down to the kidney wound. No stitches were inserted into the kidney substance. For fourteen days urine escaped by the wound, after which it healed kindly, and the patient left the hospital three weeks later cured. I have seen the man lately, and he has not lost a day's work since.

**Case 2.**—A collier, aged 27, had right lumbar pain on and off for four years. He had a history of several attacks of renal colic, with the passage of gravel. He came under my care on March 22nd, 1900. There was pus in the urine, which was acid, and there was slight hæmaturia. He stated that on occasions there was a distinct swelling in the right lumbar region. He was admitted into the Aberdare Cottage Hospital on March 23rd, and sounded for stone, with a negative result. As he was anxious for operation, I cut down on the kidney and found a stone blocking the pelvis of the kidney. An incision was made along the convex border and the stone extracted by forceps, after the exercise of considerable patience, as it would have been easier to make a direct incision on the stone through the pelvis. The kidney was then well flushed out and dressed in the usual way.

**Case 3.**—On November 10th, 1901, a man (M. J.) came to me with the following history:—For some years he had suffered from renal colic, which passed off under treatment; but lately he noticed the urine was dark and porter coloured, worse after a long day's work. But what troubled him most was the severe pain in the right lumbar region, which shot down to his thigh. On November 25th, 1901, I cut down and found a stone firmly fixed in the pelvis. After a good deal of manœuvring with scoop and forceps, I managed to extract it through the convex border of the kidney. The hemorrhage was severe, but easily controlled by pressure. The case was dressed in the usual way. He was discharged in five weeks' time, and has been in good health since. For the first week there were some distressing symptoms of colic, caused by blood clots passing down the ureter, which were relieved by large draughts of hot water.

**Case 4.**—A man, aged 30, was seen by me on January 19th, 1902, with severe colic and left lumbar pain. He had not had such pain for ten years, when he suffered acutely for about a week, when he believed he passed something which gave him great relief, and was free from pain until the present attack. He got better under treatment, but a month later I was called to see him in great pain. His urine was acid, with hæmaturia and some pus. Being anxious to be relieved, I admitted him into the Cottage Hospital, and cut down on the kidney on February 23rd, 1902, and found a small, hard stone with a pointed edge lying towards the ureter. On removing the stone through the convex border of the kidney I found a small distinct facet on it, but on carefully searching the pelvis and kidney proper could find no other stone present. It is possible that the other stone passed ten years previously, when he had the first attack. The patient made a good recovery, and has since enjoyed excellent health.

With regard to the diagnosis of stone in the kidney, the more or less fixed renal pain, plus slight recurrent hæmaturia worse after exercise, the blood being intimately mixed with the urine, but slight and dark in character, are when present no doubt diagnostic of stone. And in these cases there can be little hesitation in exploring the kidney, especially when the pain is definitely referred to one side; and in those cases of doubt the X-rays will in future play a more important part in the diagnosis, as mentioned in a paper by Mr. Hutchinson, jun., in the *British Medical Journal* for October 19th, 1901.

With regard to the pelvic stones, in two of the above cases it would have been much easier to have cut direct on the pelvis; but I was afraid of a fistula by this method, as I believe there is less risk of urinary fistulæ by cutting through the cortex, although there may be more hemorrhage.

